# Effect of Drilling Parameters and Tool Diameter on Delamination and Thrust Force in the Drilling of High-Performance Glass/Epoxy Composites for Aerospace Structures with a New Design Drill

**DOI:** 10.3390/polym16213011

**Published:** 2024-10-27

**Authors:** Bekir Yalçın, Çağın Bolat, Berkay Ergene, Uçan Karakılınç, Çağlar Yavaş, Yahya Öz, Ali Ercetin, Sinan Maraş, Oguzhan Der

**Affiliations:** 1 Department of Mechanical Engineering, Faculty of Technology, Afyon Kocatepe University, 03200 Afyonkarahisar, Türkiye; 2 Department of Mechanical Engineering, Faculty of Engineering, Samsun University, 55420 Samsun, Türkiye; cagin.bolat@samsun.edu.tr; 3 Department of Mechanical Engineering, Faculty of Technology, Pamukkale University, 20160 Denizli, Türkiye; bergene@pau.edu.tr; 4 Department of Computer Programming, Vocational School of Technical Science, Isparta Applied Science University, 32200 Isparta, Türkiye; ucankarakilinc@isparta.edu.tr; 5 Karcan Cutting Tools, Organized Industrial Site, 26110 Eskişehir, Türkiye; c.yavas@karcan.com; 6 Advanced Composite Materials Technology Center, R&D and Technology Directorate, Turkish Aerospace, 06980 Ankara, Türkiye; yahya.oz@tai.com.tr; 7 Department of Naval Architecture and Marine Engineering, Maritime Faculty, Bandırma Onyedi Eylül University, 10200 Bandırma, Türkiye; aercetin@bandirma.edu.tr; 8 Department of Mechanical Engineering, Faculty of Engineering, Ondokuz Mayıs University, 61300 Samsun, Türkiye; sinan.maras@omu.edu.tr; 9 Department of Marine Vehicles Management Engineering, Maritime Faculty, Bandırma Onyedi Eylül University, 10200 Bandırma, Türkiye; oder@bandirma.edu.tr

**Keywords:** glass-reinforced laminates, drilling optimization, thrust force, delamination, aerospace applications

## Abstract

Real service requirements of the assembly performance and joining properties of design components are critical for composite usage in the aerospace industry. This experimental study offers a novel and comprehensive analysis of dry drilling optimization for glass-reinforced, high-performance epoxy matrix composites used in aerospace structures, focusing on thrust force and delamination. The study presents a first-time investigation into the combined effects of spindle speed (1000, 2250, 4000 and 5750 rpm), feed rate (0.2, 0.4, 0.6 and 0.8 mm/rev) and tool diameter (3 and 5 mm) using a custom-designed drill tool specifically developed for this application, filling a gap in the current literature. By employing the Taguchi design of experiments, the study identified that medium spindle speeds (2250–4000 rpm), lower feed rates (0.2 mm/rev) and smaller tool diameters (3 mm) provided optimal conditions for minimizing thrust force and delamination. These results present actionable insights into improving the structural integrity and performance of drilled aerospace-grade composite components, offering innovative advancements in both the aerospace and defense industries.

## 1. Introduction

Over the last decades, general sensitivity to clean manufacturing techniques, advanced engineering materials, recycling technologies and renewable energy has reached a significant point in academic and sectoral surroundings. As such, topics of innovative engineering design and production sustainability have been evaluated as critical headlines both for real manufacturers and researchers inclined to materials science. When frontrunner industrial sectors are considered, certain areas such as automotive, aviation, construction, marine and defense can be distinguished due to their common impact on the environment and air pollution [1,2,3]. On the other hand, lately, the number of scientific efforts on fuel efficiency, lightweight materials and alternative nature-friendly fuels has increased to provide better solutions for future applications in the transportation sector. Parallel to this, correct material choice and optimized design usage for a target system component are substantially determinant subjects that immediately affect the total mass of the design, average fuel consumption, released harmful gases and utilized energy during service conditions [2,3,4]. In light of these kinds of necessities and objectives, fiber-reinforced laminate composites have emerged as promising materials in the automotive, aviation and aerospace industries, thanks to their high specific strength, sufficient stiffness and low density [5,6]. In addition, some of the fiber-added polymer matrix laminates are suitable for secondary operations such as heat treatment and UV radiation, thereby obtaining better mechanical performance [7,8].

Considering the present literature and ongoing industrial projects, it can be seen that there are many different matchings for reinforcement/matrix variations of laminated polymer composites. At this point, performance-based and component-oriented optimization endeavors are ongoing by various investigation teams. If reinforcement materials are analyzed, in comparison with other options, glass and carbon fibers can be marked as the most preferred materials [9,10]. In addition to these fibers, basalt and natural (jute, sisal, sugar palm, coir and cotton) fibers have also been used in recent years [11,12]. Especially in terms of glass and carbon fibers, the general demand is relatively higher for the specific critical sectors like aerospace, automotive and defense due to their unique structural properties, namely relatively low density, good flexibility and sufficient rigidity. However, since these fibers have low fracture toughness and high brittleness, detailed optimization efforts are necessary on the volume fraction of fiber or filler materials to attain the best tensile strength, flexural strength, impact resistance and machinability [13,14]. In addition, all these works should be incorporated with comprehensive real-time service performance, energy efficiency and design cost analyses, thereby building a useful bridge between academia and present industry. As for the matrix polymers, thermoset-based resins are being investigated due to their proper fluidity, wettability under the vacuum and sufficient yield strength [15]. Although the majority of the studies are focusing on thermoset resins, matrix phases have also been examined in laminated composite works in recent years to benefit from their high elongation capacity and satisfied ductility [16].

When the existing scholarly archives are scanned meticulously, even though main project areas focus on fabrication optimization [17,18], mechanical properties [19,20], interphase analyses [21,22] and filler/matrix combinations [23,24], it can be noticed that the number of machining works of laminated polymer composites has begun to rise in the last years. Particularly for aerospace applications (wing parts and fuselage components), assembly and mechanical joining play a critical role in final products. Herein, drilling operations are critical and have to be performed with minimum delamination, maximum dimensional accuracy and minimum surface roughness. Wing and fuselage parts of the aircraft are subjected to dynamic forces and delamination cracks can play a stimulating role in the cyclic damages in the vicinity of assembly zones. On the other hand, excess surface delamination causes liquid leakage during service conditions, and localized bulging sections are formed in the joint points. Thus, the mechanical responses of the joint element have an adverse influence and service life diminishes. Moreover, minimum cutting force usage positively triggers the fabrication economy, and this study also introduces a new drill tool to minimize the thrust force levels with the minimum delamination damage on the composite laminates. The complex problem of montage, fabrication and economy in the aerospace industry can be figured out via novel tool-design and -optimization approaches specific to composite material types. As for other strategic applications requiring sensitive drilling, joining points of battery boxes of electrical cars and assembly holes of wind turbine blades can be shown. In this context, drilling process-oriented parameter solutions including cutting speed, feed rate, lubricant addition, depth of cut, tool type and tool geometry emerge as strategies. Lately, additional optimization works such as statistical routes and neural network approaches have been also integrated into the drilling of laminated polymer composites to improve the surface quality and geometrical precision.

Scientific efforts have been directed at drilling optimization, surface quality and delamination analyses of laminated polymer composites. To gain a useful practical benefit, obtained outputs have been introduced to real industries like aircraft manufacturers, rocket firms and defense system companies. For instance, Masannan et al. [25] conducted experimental studies on the drilling of the kenaf/PLA laminates and noted that measured thrust force values escalated with applied feed rates. Shetty et al. [26] studied natural fibers (coconut coir) and incorporated design of experiment (DOE) techniques with neural network to create a strong optimization approach. The research team reported that the average error percentage for thrust force and torque were 1.75 and 6.56%, respectively. Prakash et al. [27] offered to utilize the finite element technique to estimate the thrust force in the drilling of glass fiber-added polymer laminates and propounded that, due to the cutting force, the minimum level converged with the high cutting speed. Ze et al. [28] emphasized the risk of thermal damage during the drilling of laminated polymer composites owing to the different thermal conductivity of the reinforcement and matrix material. Rao et al. [29] tried the variance analysis (ANOVA) method to explore the delamination response of aramid fiber-added polymer laminates and claimed that there was an increasing trend of delamination for the decrease in speed of spindle and drill diameter. In another paper, Lukacs et al. [30] proposed an alternative technique to determine the delamination factor of the glass-reinforced laminates by the way of image differencing. Biruk-Urban et al. [31] underlined that push-out style delamination was more dominant than the peel-up style for glass fiber-reinforced epoxy laminates in the cutting speed range of 90–365 m/min. Arhamnamazi et al. [32] indicate that visual inspections should be supported via extra detailed routes like radiography to assess the delamination damage of carbon fiber-added laminates; otherwise, the correct delamination area could not be made clear. Thakur et al. [33] found that graphene addition (between 0.125 and 0.25 wt%) to the polymer matrix led to a reduction in thrust force values during the drilling of the glass-reinforced laminates. Demirsöz et al. [34] mixed glass beads and fibers in the PA66 polymer matrix to examine drilling features of the specimens. The research team asserted that thrust forces showed a rising trend of about 100–200% with higher feed rates, while a decreasing tendency of about 50–100% was found with ascending cutting speed. Upputuri et al. [35] implemented the fuzzy logic approach to predict the thrust force and delamination values of carbon fiber-filled epoxy laminates. Results indicated that the drill diameter was the most effective input variable on the delamination factor. Similarly, in recent years, parallel applications and artificial neural network-based solutions have also been utilized to ascertain the optimum drilling parameters by other investigation groups [36,37,38]. Apart from these contributions, some articles focus on cutting tool technologies to save drilling energy and drop the average thrust force levels. Xu et al. [39] compared different tool geometries in terms of wear during the drilling of carbon fiber-filled polymers and stated that candlestick tools displayed more abrasion resistance than step drills. Bolat et al. [5] found that a twist drill (120° point angle) with a 5 mm diameter was the best option for the low drilling force and delamination behavior of carbon fiber-reinforced composite laminates. Xu et al. [40] expressed that diamond-coated candlestick drills caused lower cutting zone temperatures than step drills in the drilling of polymer matrix laminates.

In this paper, unlike previous studies in the literature, glass fiber-reinforced high-performance tough resin matrix composites were utilized and the combined influence of drill tool diameter, feed rate and cutting speed on thrust force levels and delamination factor were examined experimentally for the first time. A novel, specially designed drill tool was used in this study, offering a unique approach to optimizing drilling performance. To simulate the design need of the real sector, drill diameters were altered for the aerospace-grade laminates and this input was merged with the main cutting variables to explore their total effects on the cutting energy. The principal goal of this effort is to optimize the geometrical inputs and process to achieve the lowest delamination damage and minimal thrust force, which is critical for improving the assembly performance and economic efficiency in the aviation sector.

The primary objective of this study is to optimize drilling parameters, including spindle speed, feed rate and tool diameter, for aerospace-grade glass fiber-reinforced epoxy composites to minimize thrust force and delamination. By utilizing the Taguchi design method and a newly designed drill, this study aims to identify the best parameter combinations that improve the mechanical integrity of the drilled composite components, a contribution not yet addressed in the literature for these specific materials and conditions. The results from this investigation are expected to contribute to advancements in the aerospace industry by providing insights into more efficient and reliable drilling processes for composite materials.

## 2. Materials and Methodology

### 2.1. Materials

HexPly^®^ 8552 composite laminates with dimensions of 43 × 30 cm^2^ with a multi-layered rigid structure were drilled in this work. This composite is an amine-cured, toughened epoxy resin system supplied with 7781 E-glass fibers with a dry fabric weight of 299 g/m^2^ and a fabric thickness of 0.22 mm for use in aerostructures. The epoxy has a density of 1.301 g/cm^3^ and a wet (dry) glass transition at 154 (200) °C. The prepregs, which have a resin content of 37%, exhibit good lightweight, high strength, stiffness and resistance to environmental factors such as corrosion and fatigue for a wide range of applications. Preparation of the composite was performed by uniform hand lay-up of 14 plies of the described unidirectional prepregs. Note that a sequence of 14 plies is commonly used in a number of aerostructures of civil aerospace platforms. The stacking sequence was 45°, 0°, 90°, −45°, 90°, 0°, 45°. The resulting laminate has a thickness of 3.36 mm. The plies of the composite were vacuum debulked to the mold at 20 °C during the hand lay-up to remove trapped air. Subsequently, autoclave curing was performed. Curing conditions of the composite were 7 bar as gauge autoclave pressure, 180 ± 5 °C curing temperature, 120 min dwell time and 3 °C/min heat up and cool down rate. In essence, these prepregs are commonly used in the aerospace industry to manufacture advanced composite structures. They are favored for their lightweight properties and high strength-to-weight ratio. For instance, the material can be used for cabin panels, seating components and other interior parts, wing panels, fuselage sections and tail assemblies where lightweight and fire resistance are beneficial. The resulting composite material exhibits outstanding mechanical properties, including high resistance to impact and excellent fatigue performance. These attributes are critical for aircrafts, where components must withstand significant stresses during operation and under varying environmental conditions. Moreover, the lightweight nature of the composites contributes to overall fuel efficiency. By reducing the weight of aircraft components, this material helps to decrease fuel consumption and lower emissions, aligning with the aerospace industry’s goals for sustainability and cost-effectiveness. Thus, this high-performance material is pivotal in modern aircraft construction due to its exceptional properties, which enhance both safety and efficiency in flight operations. Hence, these composites are used in aircrafts like the Boeing 787-10 Dreamliner (Boeing, Arlington, VA, USA), Airbus A350-1000 (Airbus, Haute-Garonne, Toulouse, France), Gulfstream G800 (General Dynamics, Chantilly, VA, USA), Cessna Citation Latitude (Textron, Providence County, RI, USA) and Bombardier Global 8000 (Bombardier Inc., Montreal, QC, Canada). In the automotive sector, lightweight panels for cars and trucks can be manufactured to improve fuel efficiency in addition to structural components like frames, chassis components and suspension parts where stiffness and strength are critical. Frames and components for bicycles and other sports equipment like tennis rackets, golf club shafts and other sporting gear, where lightweight, stiffness and impact resistance are important, can also be produced with the material. These prepregs are also used in the construction of boat hulls and decks due to their resistance to water and high strength. Sailboat masts and other structural components are also of relevance due to their light weight and stiffness. The material is also used in the construction of wind turbine blades due to their high strength and stiffness, which can withstand forces encountered in wind energy applications.

### 2.2. Machining Procedure and Taguchi Analyses

**Figure 1:**
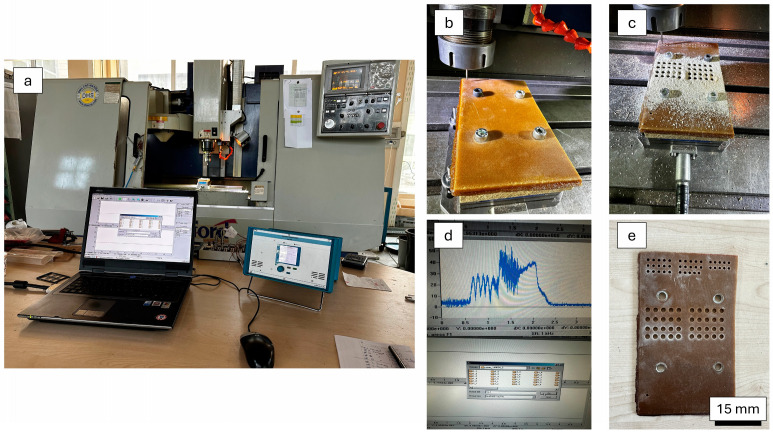
(**a**) Machining process images; general view of the CNC system; (**b**) clamping of the laminated samples and dynamometer fixing; (**c**) drilling operation; (**d**) data collection with special software; (**e**) drilled view of the final product.

**Figure 2:**
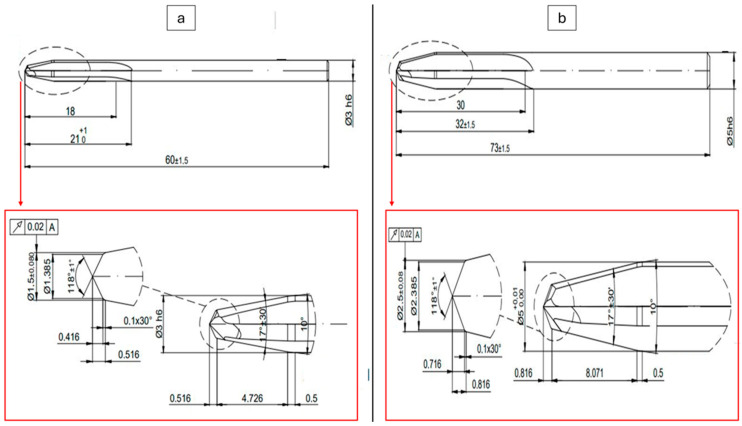
Technical drawings of the utilized drill tools; 3 mm (**a**) and 5 mm (**b**).

Drilling processes were practiced at Süleyman Demirel University Innovative Technologies Center (YETEM), Isparta, Turkey. A computer numerical control (CNC) operation center (Hartford VMC 1020, Taichung City, Taiwan) was run for the drilling of composites manufactured by taking required security precautions. As evidenced by prior research in the literature [41,42], the utilization of support structures during drilling operations tends to result in reduced thrust force values. Prior to the drilling tests, a wooden plate was positioned between the fabrication table and the composite specimens. The fixation process was then conducted via fasteners, as illustrated in Figure 1b. To collect the thrust force data, a computer-controlled Kistler 9257B digital dynamometer (Winterthur, Switzerland) was integrated into the CNC equipment. This dynamometer runs between −5 kN and 10 kN as force interval with data sampling frequency of 5000 s^−1^. Drill tools utilized in this work were supplied from the Karcan Cutting Tools Company (Eskişehir, Turkey). In Figure 1 and Figure 2, the real-time machining image of the laminated composites and geometrical details of the utilized drill tool can be seen, respectively. In addition to the 118° tip angle, the new design tool, illustrated in Figure 2, features two distinct angles resulting from the cross-sectional alteration, a distinctive attribute not observed in other tools. These angles are specifically configured to facilitate the cutting of fibers.

In order to produce applied knowledge, macro drilling experiments of the selected glass-reinforced polymer-based composite, which is frequently used in aviation applications, were carried out. Drilling tests were designed by the Taguchi method to obtain the optimal parameter combination through the calculation and analysis of experimental data. It is known that the Taguchi method requires following these steps: (a) identify the problem and objectives of the experiment; (b) select quality characteristics and measurement systems; (c) select input factors and their level; (d) choose the appropriate orthogonal arrays (OAs) and determine the experimental plan; (e) conduct experiments and record the experimental data; (e) analyze experimental results, e.g., using S/N analysis, factor effect, DOE and ANOVA. In this study, all these requirements are met with input variables as follows: spindle speeds (1000, 2250, 4000, 5750 rev/min of four levels), second feed rates (0.2, 0.4, 0.6, 0.8 mm/rev of four levels) and finally drill diameter (3 and 5 mm of two levels) are determined. Lower spindle speeds are investigated for larger drills due to the tough composite to reduce heat generation and avoid damaging the material. Higher feed rates are being studied to increase the drilling speed. Smaller drill diameters are used for precision drilling. To summarize, parameters and their levels were determined based on existing literature and the capabilities of the equipment used [43,44,45,46].

In particular, the problem definition in drilling fiber-reinforced laminated composites is to establish the relationship between thrust force and delamination in this study. Therefore, thrust force and delamination measurements were achieved for each drilling condition with five repetitions and results were averaged. The Taguchi experimental plan designed with L16 orthogonal array shows input variables in Table 1. The signal-to-noise ratio (SN), main effect, interaction plot and contour plot diagrams and response table for SN and model summary were obtained from Taguchi and Taguchi DOE analyses. The SN ratio is then analyzed to identify the preferable parameter values with the best factor levels. The parameter with the most impact on thrust force and delamination is determined with all Taguchi analysis results. As can be seen in Table 1, drilling tests for aerospace grade glass-reinforced polymer-based composites were performed. In experimental design, mixed level design was preferred because the input parameter levels were not the same. Four levels of spindle speed, four levels of feed rate and two different drill tool diameters were tried to determine the effect of drilling parameters on the thrust force and delamination. The “smaller is better” function was chosen for Taguchi to analyze thrust force and delamination outputs.

On the way to real assembly applications of laminated composites, delamination is a considerably vital issue affecting the target performance of design components during actual service conditions. Different groups have used some mathematical approaches to assess the delamination. The delamination factor term is determined to explore the magnitude of the drilling damage-based separation between composite plies. Despite the presence of other formulas, in general, after the dry drilling of laminated composites, the delamination factor at the tool exit side is calculated with the ratio of the highest diameter covering damaged sections to the nominal diameter. This methodology was also adopted in this work and damaged sections were analyzed with the combination of Leica S8AP0 (Wetzlar, Germany.) model stereo zoom microscope and image-processing software (Image J 1.53).

## 3. Results and Discussions

### 3.1. Thrust Force Analyses

**Figure 3:**
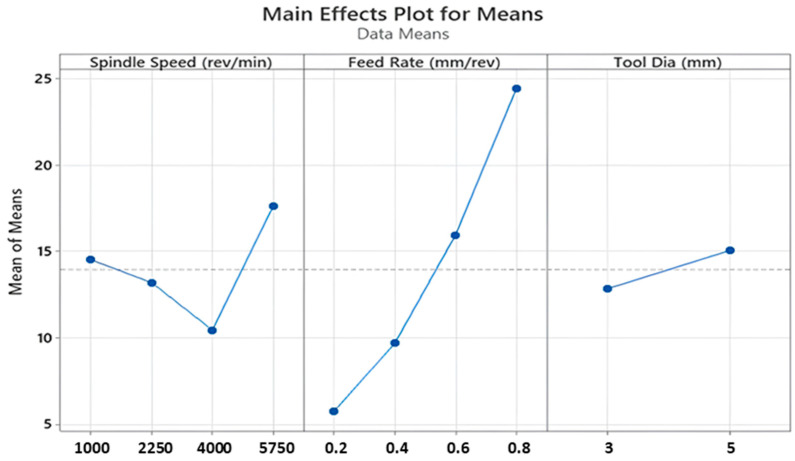
Main effect plots for thrust force values.

**Figure 4:**
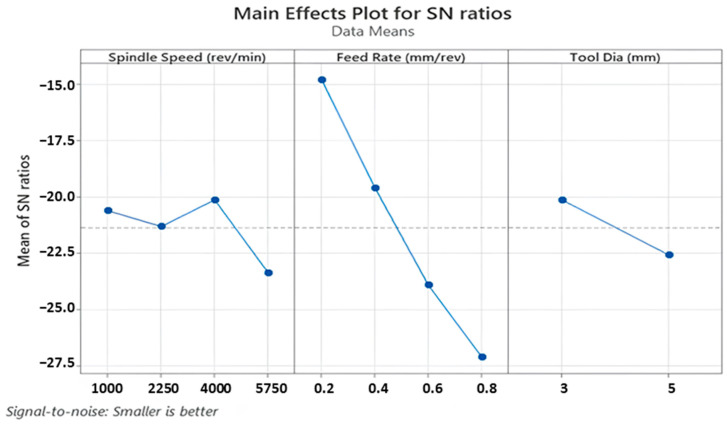
SN ratio plots for thrust force values.

**Figure 5:**
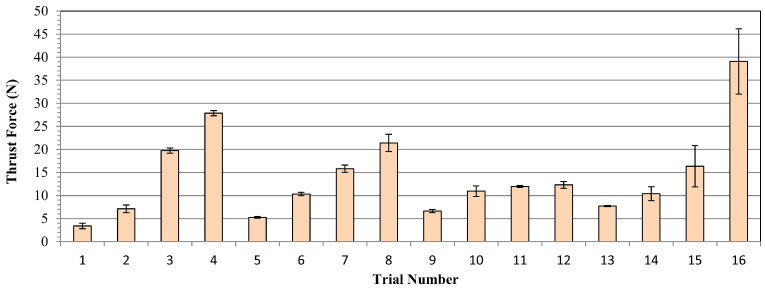
Average results of Taguchi trials for thrust force values.

Thrust force is a fundamental term that impresses directly on the cutting force of the drill tool during hole-making operations. Also, it affects hole quality, tool wear and consumed energy throughout the machining. On the other hand, it depends strongly on cutting parameters like feed rate, cutting speed and depth of cut. According to Taguchi results obtained from the experiments, the main effect and SN ratio plots of the measured values can be seen in Figure 3 and Figure 4 below. In addition, all trials can be monitored in Figure 5. SR, SqR and Sq(adj) values of the model applied for Taguchi analysis for drilling force were obtained as 4.2151, 96.38% and 84.60%, respectively. On the other hand, these values were obtained as 3.62, 96.38% and 79.42%, respectively, in Taguchi analysis for delamination. These values are known to be at acceptable rates in the literature [47].

The highest thrust force value of 39.1 N was detected for the sample drilled with the highest feed of 0.8 mm/rev. With the lowest feed of 0.2 mm/rev, the lowest force of 3.4 N was recorded. Furthermore, a review study on the drillability of laminated composites [48] included an analysis of critical thrust force values as a function of the tool types employed. It is asserted that thrust forces below the critical thrust value will result in a more favorable delamination outcome. The critical thrust forces for the various tool types (twist drill bit, slot drill bit, brad point drill bit, core drill bit and step drill bit) were found to be approximately between 25 N and 45 N. Given that the majority of the thrust force values obtained from this experimental study are below 25 N, with the remaining values falling between 3.4 N and 39.1 N, it can be concluded that the majority of the thrust force values fall within the range of critical thrust force values. From Figure 3 and Figure 4, it can be noticed that there is an affirmative relation between increasing feed rate and detected increasing thrust force values. This case can be expressed by the diminishing step number on the cutting height of the sample, which leads to wider contact surfaces between drill tool and workpiece surface in one step. Broadening the contact area leads to bigger plastic-deformation sections on the workpiece samples by triggering cutting energy. On the other hand, depending on the production uniformity, the probability of facing difficult-to-cut glass fibers on the tool penetration surfaces increases with the escalating feed rates. For instance, Malik et al. [49] investigated the influence of the feed rate on the thrust force during the drilling of glass fiber-reinforced polymer (GFRP) composites. Their findings indicated that thrust forces increased significantly with the increase of feed rates. In another study, Mudhukrishnan et al. [50] examined the interaction between drilling parameters and thrust force for GFRP composites. The research group of Mudhukrishnan observed that an incline in the feed rate from 0.05 to 0.25 mm/rev resulted in an increase in the average thrust force value from 33.13 N to 74.12 N, while maintaining a constant set of drilling parameters.

**Figure 6:**
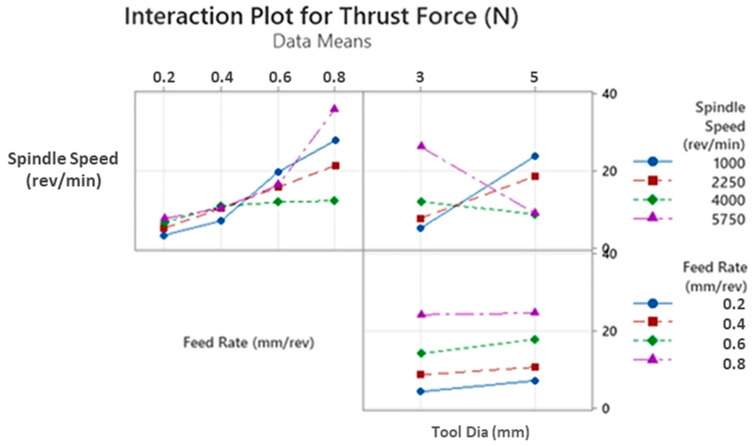
Interaction plots for thrust force values.

For this kind of aerospace-grade laminate, drill tools with lower diameters can be selected to lower the thrust force levels, so 3 mm tools are advantageous compared to 5 mm versions. As also followed in the interaction plots in Figure 6, apart from the highest spindle speed of 5750 rev/min, higher tool diameters entail the rising of cutting forces for all feed rates and spindle speeds. These outcomes can be explained by broad contact surface areas between cutting edges of the tool and the laminated workpiece. At the highest spindle speed, the tool penetrates at the centerline of the cutting geometry by way of mechanical indentation, and the workpiece material is pressed to the circular corners of the drill edges. For the small drill diameters, the indentation-based movement is relatively easy at the 5750 rev/min speed and it causes material transportation to the inclined cutting edge of the drill; thereby, the cutting force gains an upward tendency. Similar observations were also made by other research teams focusing on dry drilling of polymer laminated composites. In a study conducted by Latha et al. [51], the effect of drill geometry on the thrust force during the drilling process of GFRP was determined. The study found that an increase in drill diameter from 6 to 10 mm led to an increase in thrust force for all types of drill bits, including multi-facet, brad and spur, in addition to step drills. Furthermore, El-Sonbaty et al. [52] and Mohan et al. [53] conducted a performance analysis on the drilling performance of GFRP, which indicated that thrust forces increased with the inclination of the drill diameter due to an increase in the shear area.

As for the effect of the spindle speed, there is not a general rising/decreasing trend between thrust force and spindle speed, which can be accounted for by the altering deformation mechanism of the workpiece surface and tool material cutting edges. That kind of inclination was also found in the previous literature. In a recent study, Rajamurugan et al. [54] examined thrust forces involved in drilling GFRP composites. They employed the response surface methodology to analyze the data and found that thrust force values fluctuate with changes in spindle speeds. These findings align with results reported in this study.

**Figure 7:**
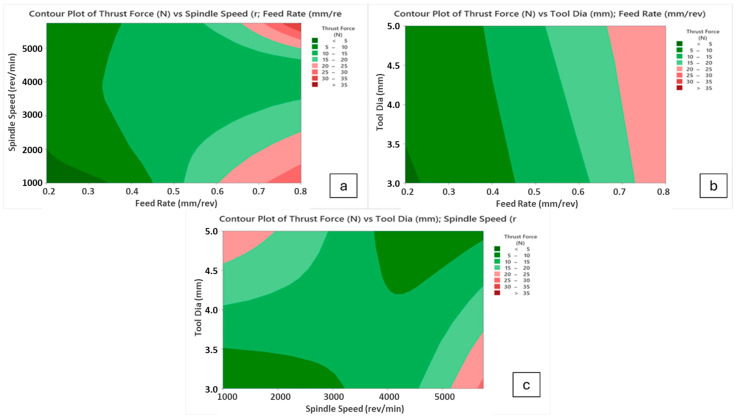
Contour diagrams for thrust force values; (**a**) spindle speed/feed rate, (**b**) tool diameter/feed rate, (**c**) tool diameter/spindle speed.

In this paper, considering main effect plots, increasing spindle speed values causes thrust force reduction but the case changes again when the spindle speed reaches the peak value of 5750 rev/min, especially for higher feeds. Contour diagrams given in Figure 7 support and justify this observation. Up to 4000 rev/min spindle speed, thermal softening mechanisms on the polymer composite laminates are effective at the vicinity of the cutting zone, thereby lowering average cutting forces. When the change of this decreasing trend is evaluated for 5750 rev/min, very fast cutting speed triggers high deformation rates on brittle fiber phases and bending effects of the tool material on the laminated samples emerge. Considering all outcomes taken from the Taguchi analyses, 4000 rev/min spindle speed, 3 mm tool diameter and 0.2 mm/rev feed rate levels are the most suitable and optimum parameters for the lowest thrust force values in the drilling of laminated samples.

### 3.2. Delamination Factor Analyses

**Figure 8:**
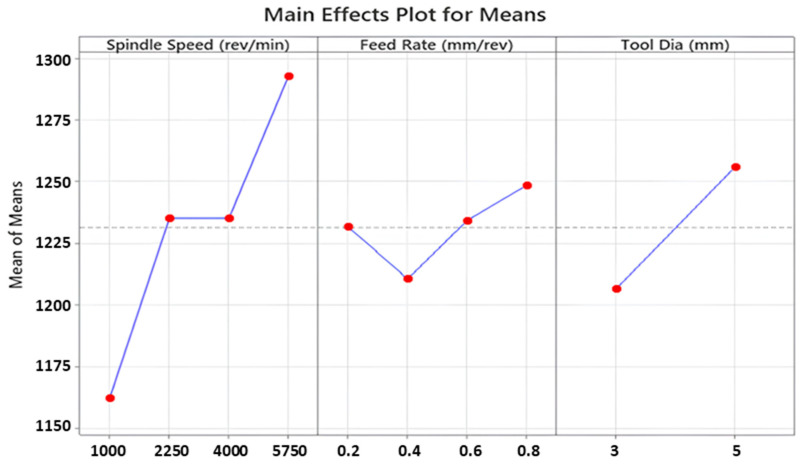
Main effect plots for delamination factor values.

**Figure 9:**
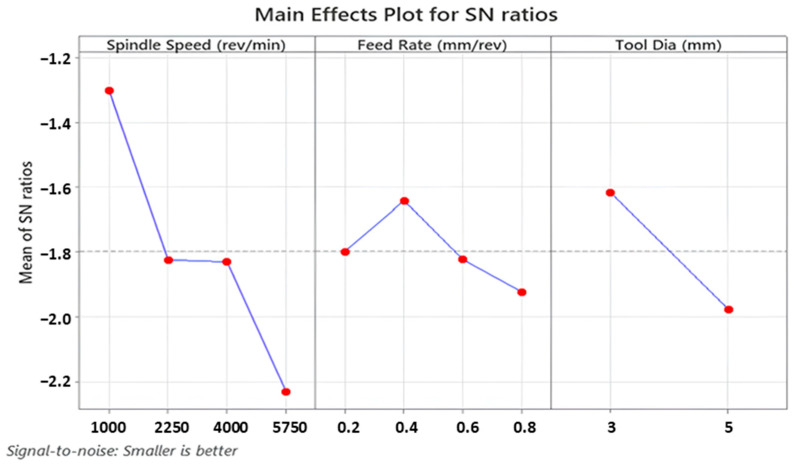
SN ratio plots for delamination factor values.

**Figure 10:**
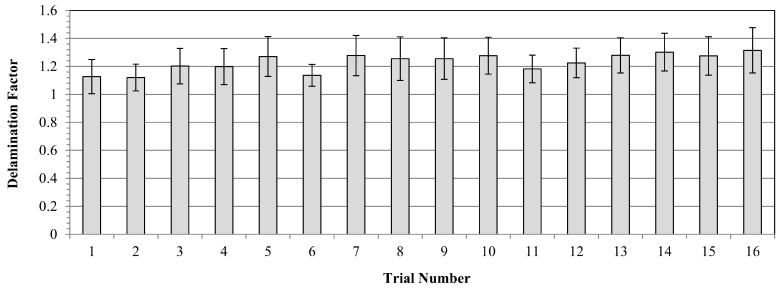
Average results of the Taguchi trials for delamination factor.

Figure 8 and Figure 9 illustrate main effects and SN ratio plots of measured samples for all three input variables. Furthermore, all average results for each Taguchi trial can be seen in Figure 10 in detail. Looking at obtained outcomes, measured delamination factor values altered between 1.121 and 1.315 depending on input combinations. Analyzing the interval, it can be evaluated as a low and low–medium segment compared to previous technical studies. Considering the outcomes, low spindle speed, medium feed and small tool diameter is the most suitable combination to reduce the delamination factor values. Additionally, Abhishek et al. [55] reported similar findings following their investigation into the drilling of CFRP with varying drill diameters (6, 8 and 10 mm). They determined that the lowest delamination factor was achieved when the smallest drill diameter was utilized during experiments. This was attributed to the fact that an increase in the drill diameter leads to an increase in the contact area of the drilled hole, which subsequently results in an increase in the thrust forces.

If the influence of the tool diameter on the delamination is evaluated, it can be seen that drill tools possessing smaller diameters are prone to lead to lower delamination factors. This circumstance might stem from the decreasing contact area between tool and workpiece. As the contact sections become bigger and wider, the probability of tool/workpiece contact on weak sections increases, and the plastic deformation-based indentation effects on the center of the drilling geometry escalate in favor of undesired bending movement of each laminate.

Spindle speed is another input that alters the calculated delamination factor values and also makes an impact on the cutting energy. From the Taguchi results, lower spindle speeds are more useful for better delamination behavior in the drilled samples. A comprehensive analysis of the literature reveals that numerous studies have been conducted to investigate effects of spindle speed on the delamination factor. These studies conclude that spindle speed can have a positive, negative or no notable effect on the delamination. For example, Davim and Reis [56] and Krishnaraj et al. [57] observed an increase in delamination with an increase in spindle speed. Conversely, Palanikumar [58] and Tsao [59] reported a decrease in the delamination factor with an increase in spindle speed. In addition to the aforementioned studies, Abrao et al. [60] and Shyha et al. [61] observed that there were no discernible effects on delamination with changes in spindle speed.

**Figure 11:**
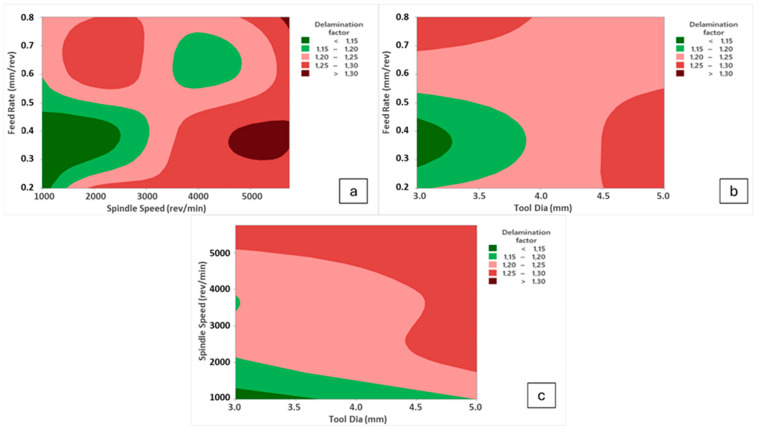
Contour diagrams for delamination factor values; (**a**) spindle speed/feed rate, (**b**) tool diameter/feed rate, (**c**) tool diameter/spindle speed.

At higher speeds, as also stated in the thrust force analyses, drill tool movement becomes more difficult due to rising cutting forces and poor evacuation of chip particles. This mechanism brings about weak delamination resistance and elevates measured delamination factor values. On the other hand, at medium speed levels, intermediate results were found when results were checked and binary interactions (Figure 11) were more significant.

As for the effect of feed rate, the highest delamination factor of 1.315 was detected for the highest feed level of 0.8 mm/rev in the 16th trial, whereas the lowest value of 1.101 was recorded for the minimum feed of 0.2 mm/rev in the 1st trial. However, it is not easy to set a direct increasing/decreasing relation between feed rate and delamination factor. Herein, for the optimum result, 0.4 mm/rev feed was determined from the main effect plots while it was a medium level among the inputs. Similar findings on fluctuation style behaviors of feed/delamination matchings were also noted in the existing literature. In consideration of the reviewed studies, it can be proposed that an increase in feed values may contribute to an increase in delamination. This phenomenon may originate from a punching process rather than a pure cutting process. In addition, Krishnaraj et al. [57] stated that lower feed rates reduced the thrust force and push-out delamination. Melentiev et al. [62] posited that a low feed rate may elevate the heating level of the surrounding matrix material, potentially resulting in matrix softening or thermal degradation. With regard to tool wear, it is advisable to select an intermediate value of feed, given that an increase in feed rate results in a reduction in contact time between the cutting tool and the workpiece material. Binary interactions indicate that high spindle speeds and medium feed rates stimulate excess delamination while low spindle speeds and medium feed rates are ideal solutions for the delamination problem. Further, the smaller tool diameter/higher feed rate and the larger tool diameter/lower feed rate combinations are the worst options for delamination.

**Figure 12:**
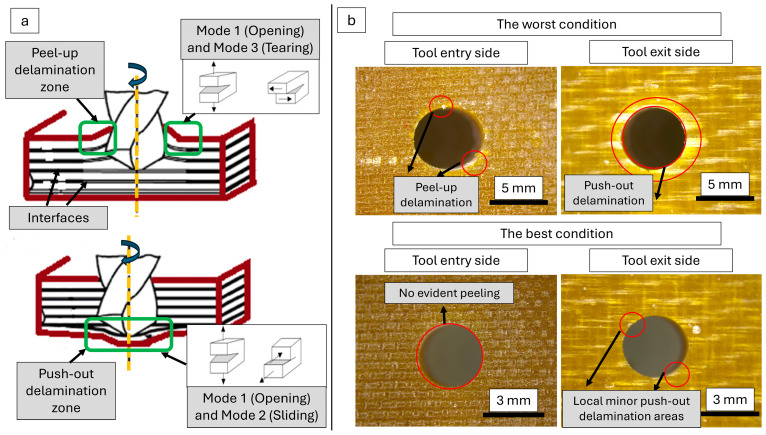
(**a**) Schematic views of the probable damage modes, (**b**) delamination analyses of the drilled samples according to the best and worst conditions.

When the existing literature is evaluated in depth, it is noticed that there are two fundamental damage modes on drilled laminated samples: peel-up and push-out delamination. Peel-up delamination occurs at the exit side with the mechanisms of mode-1 (opening) and mode-3 (tearing) while the push-out delamination emerges at the exit side of the drilled sample with mode-1 and mode-2 (sliding) mechanisms. In addition, the bending plastic deformation is also effective in the push-out style damage. According to the obtained results, the most successful sample surfaces and the worst version are compared in failure analyses to understand the delamination modes in detail as in Figure 12.

Mechanical properties like yield strength, flexural strength and impact endurance of the laminated composites alter with the sequence order of the plies, reinforcement material type and hybridization of the fillers [63,64,65]. These properties determine the damage mechanism of the composites, thereby affecting the circularity error, lateral surface roughness and material-removal rate values. In this work, a special-class aviation material of multiple plied HexPly^®^ 8552 was used to provide better suitability for the aeronautical application potential with acceptable delamination factor levels.

The outcomes indicated that the double effect of peel-up and push-out delamination could be distinguished for the sample with the highest delamination factor. In contrast, almost perfect holes were machined for samples with the lowest delamination factor. In the best combination, there were no uncut fiber marks on the tool entry surface and only a few small, localized bulged points on the tool exit side. The most apparent signs of the peel-up style delamination are spike-like individual uncut fibers and regional peeling areas. As a result of the detailed evaluations, the damage risk goes up with the climbing input parameter levels. On the other hand, the push-out style delamination needs to be blocked to obtain high montage performance under the service loads. The tool design used in this paper helps maintain good surface integrity during the dry drilling of aerospace-grade laminates.

## 4. Conclusions

The present study, which focused on the drilling characteristics of aerospace-grade laminates, revealed that thrust force levels could be reduced by employing lower feed rates (0.2 mm/rev) and smaller tool diameters (3 mm). A moderate spindle speed of 4000 rev/min was identified as an optimal parameter for enhancing tool longevity and reducing cutting forces. At higher speeds (5750 rev/min), larger tool diameters and lower feed rates were observed to be more effective than other combinations. At the lowest spindle speed (1000 rev/min), the feed rate had a more pronounced impact, particularly at 0.4 mm/rev and above. The results regarding thrust force are consistent with those of previous studies. This research indicates that the optimization of cutting conditions and tool performance through the application of experimental design techniques can assist in addressing assembly-related challenges that are unique to aerospace-grade components. Delamination, a prevalent phenomenon in drilling laminated composites, was observed to occur contingent on the selected cutting parameters. Both push-out and peel-up types of delamination were observed during the investigation. The most effective approach for minimizing delamination was found to be the use of the slowest spindle speed (1000 rev/min), a medium feed rate (0.4 mm/rev) and the smallest tool diameter (3 mm). At higher spindle speeds (5000–5750 rev/min), the majority of tool diameters exhibited an increase in delamination. However, this trend reversed at lower spindle speeds (1000–2000 rev/min) in conjunction with low to medium feed rates (0.2–0.4 mm/rev). Furthermore, combinations of small tool diameters with high feed rates and large tool diameters with low feed rates were found to increase the risk of delamination and negatively impact the quality of the drilled surface. Future research could further explore the drillability of these composites using finite element analysis. Additionally, the influence of drilling parameters and the ratio of additives could be examined with the aid of artificial neural networks or machine learning.

## Figures and Tables

**Table 1:** Taguchi Orthogonal Array Design-L16 (4^2 2^1) for the macro drilling experiment of the selected glass-reinforced polymer-based composite.

Trial No.	Spindle Speed (rev/min)	Feed Rate (mm/rev)	Tool Diameter
1	1000	0.2	3
2	1000	0.4	3
3	1000	0.6	5
4	1000	0.8	5
5	2250	0.2	3
6	2250	0.4	3
7	2250	0.6	5
8	2250	0.8	5
9	4000	0.2	5
10	4000	0.4	5
11	4000	0.6	3
12	4000	0.8	3
13	5750	0.2	5
14	5750	0.4	5
15	5750	0.6	3
16	5750	0.8	3

## Data Availability

The original contributions presented in the study are included in the article, further inquiries can be directed to the corresponding authors.
